# Bleeding management in computed tomography-guided liver biopsies by biopsy tract plugging with gelatin sponge slurry

**DOI:** 10.1038/s41598-021-04155-1

**Published:** 2021-12-30

**Authors:** Nikolaus A. Handke, Dennis C. Koch, Eugen Muschler, Daniel Thomas, Julian A. Luetkens, Ulrike I. Attenberger, Daniel Kuetting, Claus C. Pieper, Kai Wilhelm

**Affiliations:** 1 Department of Radiology, Johanniter-Hospital Bonn, Bonn, Germany; 2 Department of Radiology, University Hospital Bonn, Bonn, Germany

**Keywords:** Metastasis, Cancer imaging

## Abstract

To evaluate the safety and impact of biopsy tract plugging with gelatin sponge slurry in percutaneous liver biopsy. 300 consecutive patients (158 females, 142 males; median age, 63 years) who underwent computed tomography-guided core biopsy of the liver in coaxial technique (16/18 Gauge) with and without biopsy tract plugging were retrospectively reviewed (January 2013 to May 2018). Complications were rated according to the common criteria for adverse events (NCI-CTCAE). The study cohort was dichotomized into a plugged (71%; n = 214) and an unplugged (29%; n = 86) biopsy tract group. Biopsy tract plugging with gelatin sponge slurry was technically successful in all cases. Major bleeding events were only observed in the unplugged group (0.7%; n = 2), whereas minor bleedings (4.3%) were observed in both groups (plugged, 3.6%, n = 11; unplugged, 0.7%, n = 2). Analysis of biopsies and adverse events showed a significant association between number of needle-passes and overall (*P* = 0.038; odds ratio: 1.395) as well as minor bleeding events (*P* = 0.020; odds ratio: 1.501). No complications associated with gelatin sponge slurry were observed. Biopsy tract plugging with gelatin sponge slurry is a technically easy and safe procedure that can prevent major bleeding events following liver biopsy.

## Introduction

Tissue sampling by computed tomography (CT)-guided core biopsy is becoming increasingly important in personalized cancer therapy^1–3^. The liver is one of the most common targets of tissue sampling in clinical practice due its involvement by primary or secondary tumors as well as diffuse liver diseases^4,5^. Although the procedure is generally safe and efficient, severe complications may occur in some cases. Apart from pain, transient bacteremia, biliary peritonitis, and pneumothorax, bleeding is one of the most frequent complications^6,7^. Although the risk of major bleeding events is low (< 1%), it is increased in cancer patients (1.6%). Biopsy tract embolization with gelatin sponge can be performed in an effort to induce local hemostasis to reduce the risk of bleeding and has been described especially in patients with bleeding tendencies^1,8,9^. Gelatin sponge is completely absorbed over time and can be applied in different forms (as a slurry or in form of a so called *torpedo*) to occlude the puncture tract^8,10^. As personalized cancer therapies increase, the demand for tissue sampling and thus the risk of bleeding events, albeit rare, may increase as well. Although widely performed^1^, data on safety and clinical efficacy of biopsy tract plugging is scarce. Therefore, we analyzed our experiences in liver biopsies with and without tract plugging.

## Materials and methods

### Study design

**Figure 1:**
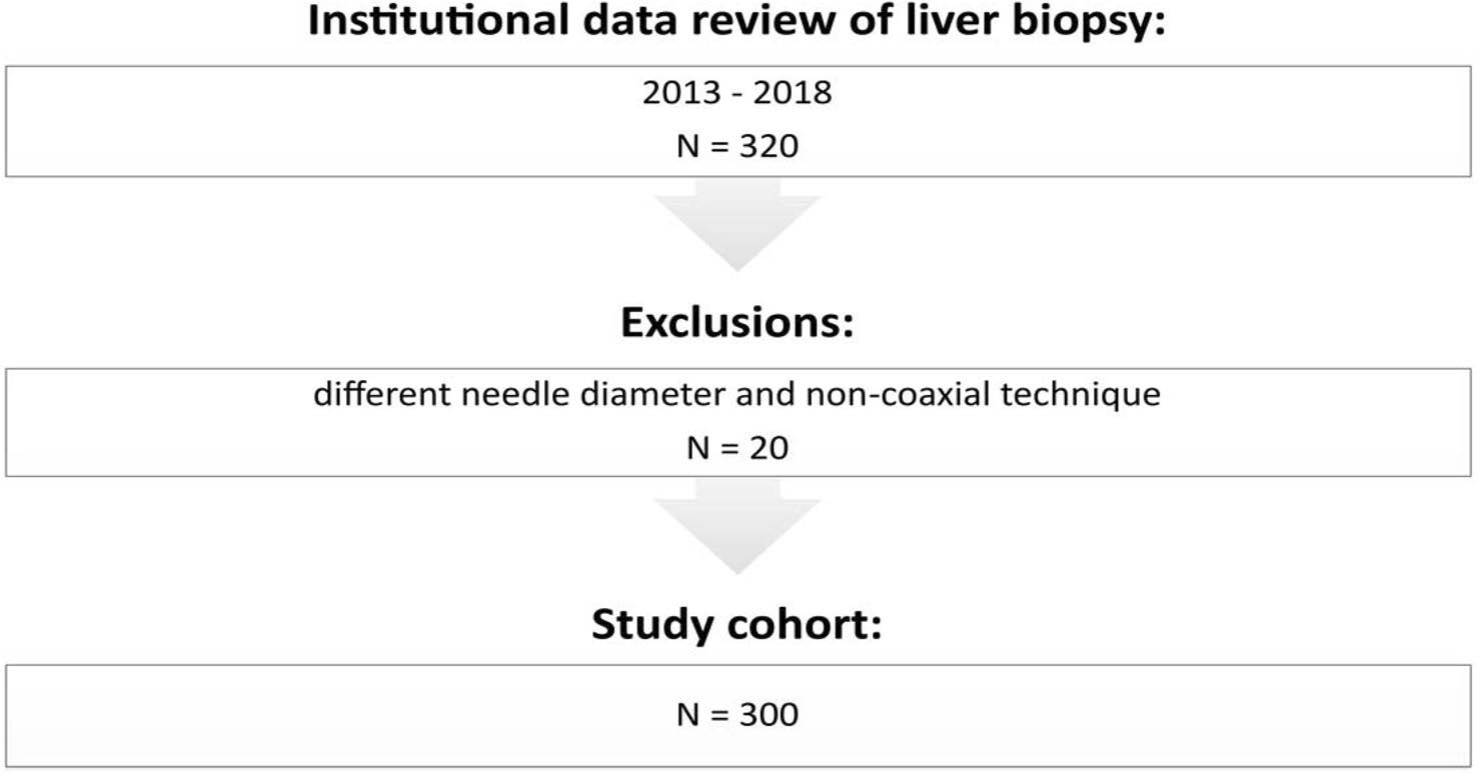
Inclusion and exclusion criteria of the study cohort.

Consecutive patients undergoing CT-guided core biopsy of the liver between January 2013 and May 2018 were retrospectively reviewed. Indications for biopsy were reached clinically in interdisciplinary consensus. All procedures performed were in accordance with the ethical standards of the institutional research committee and with the 1964 Helsinki Declaration and its later amendments. This clinical observational study was approved by the institutional ethics committee and need for written informed consent was waived (Ethikkommission Universität Bonn). Patients who underwent CT-guided core biopsy of the liver in coaxial technique using a 16-Gauge (G) core needle coaxial system in combination with an 18-G cutting needle (Cook Medical Europe Ltd., Ireland) were included in the study. Other needle diameters and non-coaxially obtained biopsies were excluded from the study cohort (Fig. 1).

### Data

Procedural parameters (diameter of the lesion (mm), localization of the lesion, biopsy tract length (mm), access route, number of biopsies) as well as histological and laboratory results [partial thromboplastin time (PTT) (s), International Normalized Ratio (INR), platelet count (G/l), Hemoglobin (Hgb) (g/dl)] and complications associated with the biopsy were recorded from the electronic in-house database of the Department of Radiology.

### Definitions

Procedures were dichotomized by application of gelatin sponge slurry into a plugged and an unplugged biopsy tract group. Postinterventional complications were assessed using the National Cancer Institute Common Terminology Criteria for Adverse Events (NCI-CTCAE) Version 5.0. NCI-CTCAE standardizes five degrees of severity of complications following medical treatment or procedure. These complications are classified as minor (CTCAE 1), moderate (CTCAE 2), severe (CTCAE 3), life-threatening (CTCAE 4) or fatal (CTCAE 5). Depending on grade of severity, a therapeutical concept is derived^11^. Bleeding was divided into minor (Hgb loss < 2 mg/dl) and major (Hgb loss > 2 mg/dl) bleedings. In case of minor bleedings, observation only was sufficient, whereas patients with major bleedings were referred to ICU observation and/or surgical/interventional treatment.

### Procedure technique

**Figure 2:**
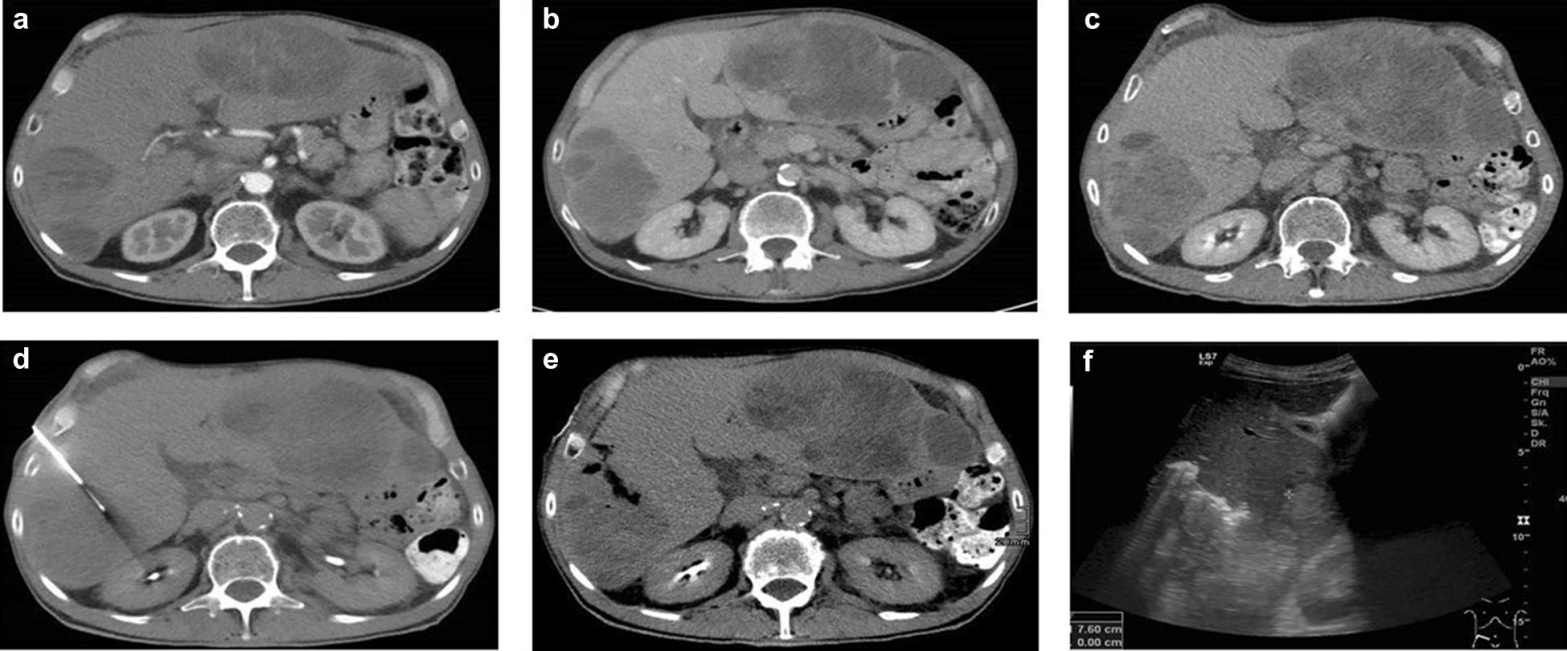
CT-guided liver biopsy and post-interventional sonography. (**a**) Axial slice of the pre-interventional CT. Target lesion in segment 5/6. Arterial phase. (**b**,**c**) Correlating axial slices of the lesion in late portal and delayed phase. Adequate biopsy tract length. (**d**) Biopsy performed with a 16-G core needle coaxial system and an 18-G cutting needle. Intralesional location. (**e**) Post-interventional non-enhanced CT. Percutaneous tract embolization by gelatin sponge slurry. (**f**) Post-interventional ultrasound. Adequate distribution of hyperechoic gelatin foam slurry. No ascites. No haematoma.

**Figure 3:**
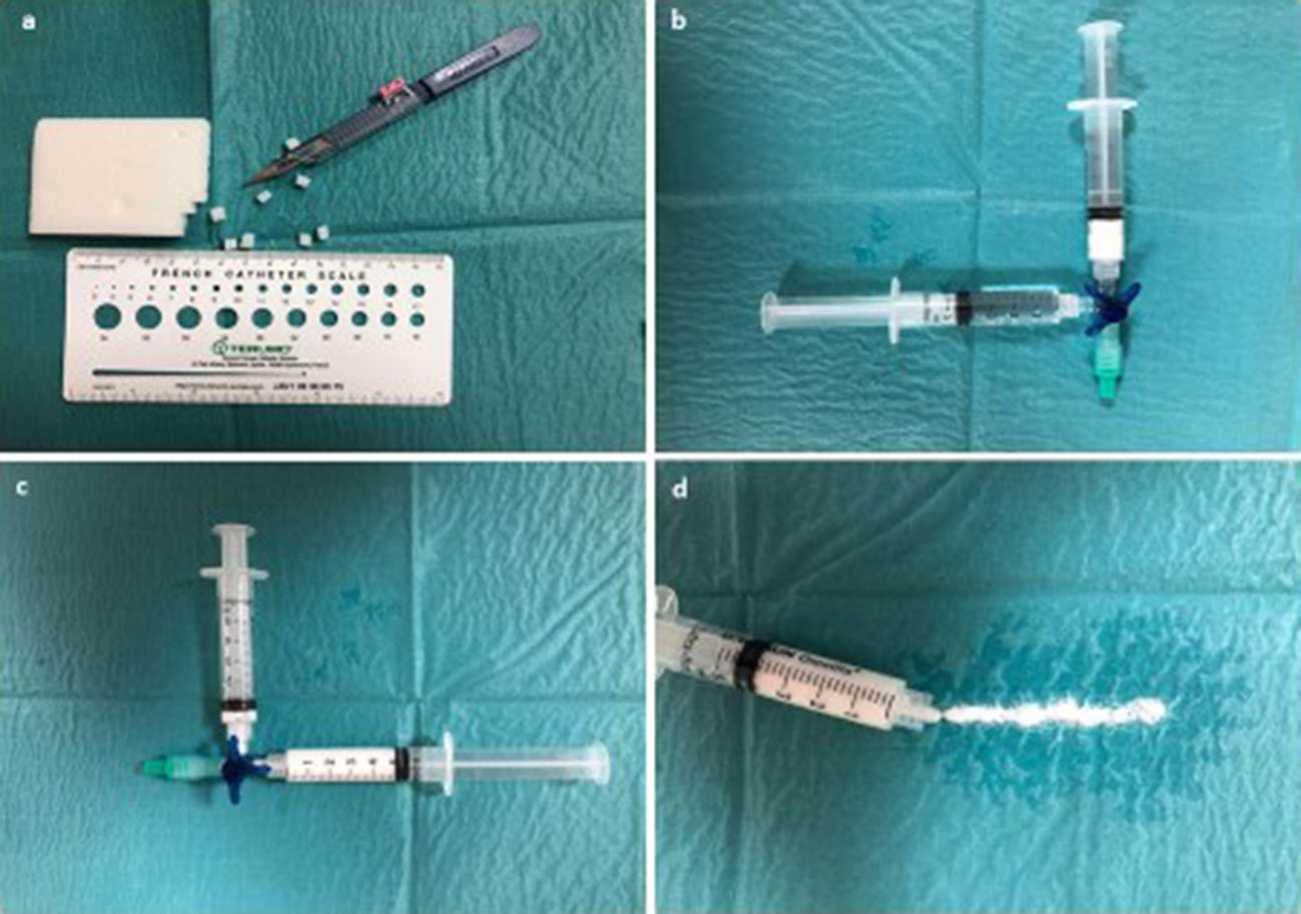
Preparation of gelatin sponge slurry. (**a**) Cutting gelatin foam in several small squares (5 x 5 mm). (**b**,**c**) Mixing 2 ml of compressed foam with 4 ml of 0.9% NaCl solution. (**d**) Viscous embolizate of gelatin sponge slurry.

Procedures were performed by experienced interventional radiologists or interventional radiology residents under direct supervision. Pre-procedural sufficient coagulation status was obligate (PTT, INR, platelet count). Otherwise, sufficient correction of coagulation parameters by platelet transfusion or tranexamic acid was performed pre-interventionally by the referring ward. All patients received a pre-interventional non-enhanced CT at the time of intervention for biopsy planning. The biopsy path was planned on an individual patient basis. Tissue sampling was performed in sterile coaxial technique under CT-guidance as previously described^1^ (Fig. 2). Specimens were preserved in formalin and sent to the department of pathology. Biopsy tract plugging was performed at the discretion of the attending interventional radiologist by using gelatin sponge slurry (CuraSpon^®^, CuraMedical B.V., The Netherlands). A 5 ml suspension of gelatin sponge slurry was prepared immediately before the biopsy procedure by cutting the gelatin sponge into small squares (5 × 5 mm) and mixing 2 ml of compressed sponge with 4 ml of 0.9% NaCl solution. Mixing the components was performed by using two 5 ml Luer–Lock disposable syringes connected via a three-way stopcock, until a viscous embolizate was prepared (Fig. 3). After exclusion of puncture or penetration of critical vascular structures (hepatic vein, portal vein branch or artery) on interventional CT images, a volume between 2–3 ml of gelatin sponge slurry was continuously applied during withdrawal of the introducer needle until it exited the skin surface after completion of the biopsy.

### Follow-up

To detect early complications (2–4 h after the procedure^12^), a post-interventional non enhanced CT was performed after completion of the procedure. Blood count control was performed within clinical course by the referring ward. Additional sonographic follow-up was ordered depending on the prior imaging and laboratory results. All biopsy procedures were performed on an in-patient basis with a post-interventional clinical follow-up of at least 24–48 h.

### Study population

In total, 300 patients were included into this study. The study cohort comprised 158 female (53%) and 142 male patients (47%). Overall age distribution ranged between 25 and 88 years (median, 63). Indications for biopsy was suspected focal liver disease in 276 (92%) and diffuse liver disease in 24 cases (8%). Detailed patient characteristics are given in Table 1.

**Table 1:** Basic characteristics of the patients and Histology (separated in categories), given as median and range or N (%).

Parameter	Valid, N	All patients	Plugged biopsy tract	Unplugged biopsy	P-value
No. of patients, N (%)	300	300 (100)	214 (71.3)	86 (28.6)	
**Sex, N (%)**					0.126
Male		142 (47.3)	96 (44.9)	46 (53.5)	
Female		158 (52.7)	118 (55.1)	40 (46.5)	
Age (years)		63.0 (25.0–88.0)	63.0 (25.0–88.0)	62.0 (27.0–87.0)	0.310
**Coagulation status**					
PTT (s)	297/212/85	28.0 (17.0–65.0)	31.0 (17.0–65.0)	26.0 (18.0–35.0)	< **0.001**
INR	295/212/85	1.0 (0.8–1.5)	1.0 (0.8–1.5)	1.0 (0.9–1.5)	0.124
Platelet count (G/l)	298/212/86	247.5 (17.0–781.0)	245.0 (17.0–781.0)	252.0 (69.0–699.0)	0.914
Hgb (g/dl)	298/212/86	12.4 (6.9–17.5)	12.4 (7.2–17.5)	12.5 (6.9–16.6)	0.779
Histology, N (%)		300	214	86	0.754
Liver cancer		46 (15.6)	31 (14.5)	15 (17.4)	
Breast cancer		40 (13.2)	29 (13.6)	11 (12.7)	
Pancreatic cancer		32 (10.6)	21 (9.8)	11 (12.7)	
Colorectal Cancer		15 (5.0)	9 (4.2)	6 (6.9)	
Lung cancer		13 (4.3)	10 (4.7)	3 (3.5)	
Other malignancy		81 (26.8)	61 (28.5)	20 (23.3)	
Benign		73 (24.5)	53 (24.8)	20 (23.3)	

### Statistical analysis

The acquisition, processing and evaluation of the underlying study data was carried out using IBM SPSS Statistics 25 (IBM Corporation, USA). Descriptive statistics were used to summarize numerical variables (median and range). Kolmogorov–Smirnov test was performed to test normal distribution of the data. For further analysis, the cohort was dichotomized in a plugged and an unplugged biopsy tract group. Group comparisons were performed using Student’s *t*-test or non-parametric Mann–Whitney *U* test. In addition, regression analysis was performed to examine the impact of risk factors on complications. Binary logistic regression analysis was used to determine the odds ratio (OR). A P-value < 0.05 was considered statistically significant.

## Results

### Procedural characteristics and technical success

The punctured liver lesions were predominantly localized in the right liver lobe (77%; n = 231; left: 21%, n = 64; bilobar: 2%, n = 5). The most frequent access route was ventral (57%; n = 171), followed by lateral (42%; n = 125) and dorsal access (1%; n = 4). Median lesion diameter was 37.0 mm (range 9.0–240.0 mm), median biopsy tract length was 40.0 mm (range 10–130 mm) and median number of obtained biopsies was 4.0 (range 1–11) (Table 2).

**Table 2:** Technical data, given as mean ± SD, median (range) or N (%).

Parameter	Valid, N	All patients	Plugged biopsy tract	Unplugged biopsy tract	P-value
Diameter of the lesion (mm)	278	37.0 (9.0–240.0)	37.5 (9.0–240.0)	37.0 (9.0–100.0)	0.118
**Localisation, N (%)**	300				0.219
Right		231 (77.0)	169 (56.3)	62 (20.1)	
Left		64 (21.3)	43 (14.3)	21 (7.0)	
Bilobular		5 (1.7)	2 (0.7)	3 (0.1)	
Biopsy tract length (mm)	300	40.0 (10.0–130.0)	42.5 (10.0–120.0)	34.0 (14.0–130.0)	**0.023**
**Access route, N (%)**	300				0.544
Lateral		125 (41.7)	85 (28.3)	40 (13.3)	
Ventral		171 (57.0)	126 (42.0)	45 (15.0)	
Dorsal		4 (1.3)	3 (1.0)	1 (0.3)	
No. of biopsies	300	4.0 (1.0–11.0)	4.0 (1.0–10.0)	3.0 (1.0–11.0)	< **0.001**

Primary technical success was achieved in a total of 285 cases (95%). In the remaining cases diagnosis was made via re-biopsy (2.3%; n = 7), clinical course (1.3%; n = 4), surgical confirmation (1.0%; n = 3) and biopsy of other tissue (0.3%; n = 1). Malignant nature of histology was more frequent (76%) than benign etiology (24%). The most prevalent biopsy result was hepatocellular carcinoma (16%; n = 47), followed by breast carcinoma (13%; n = 40) and pancreatic carcinoma (11%; n = 32).

In 214 biopsies (71%), the biopsy tract was plugged during withdrawal of the introducer needle which was technically successful in all cases. In 86 cases (29%), the biopsy tract remained unplugged.

### Group comparison

The plugged and unplugged biopsy tract group showed no significant differences in sex, histology, INR, platelet count, Hgb, diameter of the lesion, localization of the lesion and access route (*P* = 0.126, *P* = 0.310, *P* = 0.754, *P* = *0.124*, *P* = 0.914, *P* = 0.779, *P* = 0.118, *P* = 0.219, *P* = 0.544, respectively; Tables 1 and 2).

Significant group differences were found in PTT (31 vs*.* 26 s), biopsy tract length (43 vs. 34 mm) and number (4 vs. 3) of biopsies (*P* < 0.001, *P* = 0.023, *P* < 0.001, respectively; Tables 1 and 2).

### Complications

Overall complications occurred in 6.3% of cases (19/300), 0.7% of which were major bleedings (2/300) and 4.3% were minor bleedings (13/300). A pneumothorax and a colon perforation were observed in three cases (1%) and one case (0.3%), respectively (Table 3).

**Table 3:** Postinterventional adverse events and corresponding CTCAE category, given as N (%).

Parameter	Valid, N	All patients	Plugged biopsy tract	Unplugged biopsy tract	P-value
Complications, overall N (%)	300	19 (6.3)	12 (5.6)	7 (8.1)	
**Bleeding overall, N (%)**		15 (5.0)	11 (5.1)	4 (4.6)	0.829
Minor bleeding (CTCAE 1), N (%)		13 (4.3)	11 (5.1)	2 (2.3)	0.265
Major bleeding (CTCAE 3 + 4), N (%)		2 (0.7)	0 (0.0)	2 (2.3)	**0.027**
Colon perforation (CTCAE 2), N (%)		1 (0.3)	0 (0.0)	1 (1.2)	0.118
Pneumothorax (CTCAE 3), N (%)		3 (1.0)	1 (0.5)	2 (2.3)	0.151

No adverse events attributable to gelatin sponge slurry like anaphylaxis, vascular occlusion or local infections/abscesses were recorded. No postinterventional fatality was reported.

Beside clinical observation, Hgb-control was performed by the referring ward. Average pre-interventional Hgb was 12.3, average post-interventional Hgb was 12.0. In case of minor bleeding, an average Hgb-drop of 0.6, in major bleedings an average Hgb-drop of 2.2 was observed.

One major bleeding (CTCAE 4) occurred in a 56-year-old patient with a metastatic gastrointestinal stromal tumor of the jejunum (liver lesion diameter, 30 mm; localization, right lobe; access route, lateral; number of biopsies, 2; biopsy tract length, 20 mm). Postinterventional non-enhanced CT showed no complications. Laboratory diagnostics in the referring ward revealed a significant Hgb drop of 1.1 (9.0 to 7.9). The patient was immediately transferred to the surgical intensive care unit by the medical emergency team and, due to circulatory instability, emergency laparotomy was performed identifying intraperitoneal bleeding from the liver puncture tract. Surgery led to successful hemostasis.

Another major bleeding (CTCAE 3) occurred in an 81-year-old patient with metastatic prostate cancer (liver lesion diameter, 33 mm; localization, right lobe; access route, ventral, number of biopsies, 4; biopsy tract length, 39 mm). Postinterventional non enhanced CT showed no complications. The patient presented with elevated liver enzymes and cholestasis parameters. Contrast enhanced CT revealed liver hematoma and hematoma in the gall bladder. The patient was referred to the ICU for blood transfusions. No further intervention was necessary.

Both major bleedings (CTCAE, 3 n = 1; CTCAE 4, n = 1) were reported in the unplugged biopsy tract group. Minor bleedings were reported in both groups (3.6%; plugged group, n = 11, unplugged group, n = 2). The number of biopsies per patient (plugged group, 4 [range, 1–10]; unplugged, 3 [range, 1–11]) had a significant impact on overall (*P* = 0.038; odds ratio [OR] = 1.395, 95% confidence interval [CI] lower = 1.018, 95% upper = 1.913) and minor bleeding events (*P* = 0.020; OR = 1.501, 95% CI lower = 1.066, 95% upper = 2.113), but not on major bleeding events (*P* = 0.883) (Table 4). Sex (*P* = 0.811), diameter of the lesion (*P* = 0.247), biopsy tract length (*P* = 0.341) and coagulation status (PTT, *P* = 0.610; INR, *P* = 0.124; platelet count *P* = 0.815) showed no significant impact on bleeding events (Table 1).

**Table 4:** Logistic regression analysis of the cohort given as N (%), Odds ratio (OR) with 95% confidence intervals (CI).

Parameter	N	OR	95% CI	P-value
**Bleeding overall**	15			
Curaspon		0.762	0.218–2.670	0.671
Sex		0.876	0.297–2.588	0.811
Diameter		1.009	0.994–1.024	0.247
Biopsy tract		0.985	0.955–1.016	0.341
No. of biopsies		1.395	1.018–1.913	**0.038**
PTT		0.977	0.892–1.069	0.610
INR		0.989	0.978–1.001	0.972
Platelet count		0.999	0.994–1.004	0.815
Hgb		0.798	0.614–1.036	0.090
**Major bleeding**	2			
Curaspon		0.000	0.000	0.995
Sex		0.000	0.000	0.995
Diameter		0.987	0.898–1.086	0.794
Biopsy tract		0.946	0.826–1.084	0.424
No. of biopsies		0.927	0.338–2.544	0.883
PTT		0.000	0.000	0.961
INR		0.990	0.979–1.001	0.897
Platelet count		1.618	0.000	0.951
Hgb		77.936	0.000	0.980
**Minor bleeding**	13			
Curaspon		1.439	0.291–7.106	0.655
Sex		1.224	0.377–3.979	0.736
Diameter		1.010	0.995–1.026	0.181
Biopsy tract		0.988	0.956–1.021	0.465
No. of biopsies		1.501	1.066–2.113	**0.020**
PTT		0.998	0.912–1.092	0.967
INR		0.990	0.978–1.001	0.929
Platelet count		0.997	0.991–1.003	0.286
Hgb		0.804	0.606–1.067	0.131

## Discussion

Personalized cancer therapies increase demand on tissue sampling. Besides establishing the nature of a lesion, staging, therapy-monitoring, research and clinical oncological studies are based on biopsies^1–3,13^. Larger tissue volume samples on the one hand provide more adequate information about tumor architecture/microenvironment and enable multiple analyses^2,14^, but may lead to increased bleeding risk and mortality on the other^15–17^. Therefore, complication management of percutaneous biopsies is important.

Overall major complication-rate in liver biopsy ranges between 0.3 and 3.3%^18^. Thereof, rate of major bleeding events range between 0.5–1.2%, though it is elevated in tumor patients (1.6%), the female sex, and advanced age^15,19^. Procurement of more than two biopsies was reported to elevate the risk for bleeding^16,17,20^. A number of > 4 biopsies is associated with a threefold higher risk of major bleeding events^16^.

Percutaneous needle biopsy can be performed in coaxial and non-coaxial technique. Coaxial technique, as chosen in this investigation, offers several procedural advantages. Besides collection of multiple samples in a single puncture, it has been reported to reduce bleeding risk (one puncture of the organ capsule) and prevent tumor cell seeding^1^. Even though this technique introduces a larger access route, it has not been associated with higher complication rates^1,21,22^. Additionally, coaxial technique allows for tract plugging, an effective tool in bleeding management/prevention.

Compared to literature, the rate of major bleeding events in our cohort was within acceptable margins (0.7% vs. 0.5–1.2%), even though our patients demonstrated an elevated risk profile (76% tumor patients; median number of biopsies, 4 [range 1–11]; 53% females; median age, 63.0).

Minor bleedings were reported in 4.3% (plugged, n = 11/214; unplugged, n = 2/86), which is also within acceptable margins (0–10.9%)^23^. Haage and colleagues described one subcapsular hematoma and one moderate bleeding from the biopsy tract in a study of 1999 without tract plugging^24^. Thus, the self-limiting hematoma rate in their study was notably lower, although the biopsy tract was not plugged with a gelatin sponge slurry. A possible explanation for this finding might be the higher number of samples per biopsy obtained in our study cohort in comparison to Haage et al., who in general obtained only one biopsy sample for pathological diagnosis.

The number of biopsies (plugged group, 4 [range 1–10]; unplugged group, 3 [range 1–11]) had a significant impact on bleeding events (*P* = 0.038; OR = 1.395, 95% CI lower = 1.018, 95% upper = 1.913), which is concordant with the pertinent literature, where significant influence on major hemorrhage was found^16,17,20^. In comparison to Boyum et al., our study cohort was rather small (n = 300 vs. n = 5011) and risk of major bleeding events in general is low. The number of samples per biopsy did not demonstrate a significant difference regarding major bleeding events in our cohort (*P* = 0.883), but did show a correlation for minor bleeding events (*P* = 0.020; OR = 1.501, 95% CI lower = 1.066, 95% upper = 2.113).

Beside gelatin sponge slurry, biopsy tract embolization can also be achieved with other materials, e.g. embolization coils, autologous blood clots, microfibrillary collagen, or gelatin sponge in rolled form^1,9,25,26^. One advantage of gelatin sponge slurry in contrast to e.g. coils is that it is absorbed completely within weeks. Risks of dislocation, delayed local infection, or imaging artefacts associated with permanent foreign bodies can therefore be avoided^27^.

Comparison of gelatin sponge and other absorbable embolizates has so far not been performed for liver biopsy tract plugging. Gelatin sponge was compared to microfibrillary collagen for tract embolization following percutaneous transhepatic islet cell transplantation and found to be inferior^9^. However, gelatin in this study was introduced into the biopsy tract in rolled form and not mixed as a suspension. It is possible, that gelatin suspension leads to a more adequate embolization due to a more sufficient distribution than the application of rolled gelatin sponges. An advantage of gelatin sponge over microfibrillary collagen, however, is the notably lower cost (< $ 4 at our institution vs. approx. $ 180.00 in 2016). Besides these advantages, preparation of gelatin sponge slurry is fast and easy, especially in emergency cases. Severe adverse events induced by gelatin sponge slurry like anaphylaxis are rare (< 0.5%)^27,28^ and did not occur in any of our patients.

As described we performed non-contrast CT immediately after the intervention in order to detect post-procedural complications. It is arguable that post-interventional hemorrhage can also be detected sonographically forgoing the need for additional radiation exposure. However, there are inherent limitations of sonography in our patient cohort of mostly elderly, critically ill patients who were often unable to perform deep inspiration, adequate breath holds, or demonstrated meteorism. Therefore, we opted for postinterventional non-contrast enhanced CT. From a procedural point of view postinterventional non-contrast enhanced CT not only provides a basis for evaluation of complications without the need for repositioning the patient. It can also be followed by immediate contrast-enhanced CT angiography in case any complications are suspected without time loss and thus also provides the basis for adequate and immediate intervention (e.g., immediate transfer to angiography for further treatment).

This study is limited by its retrospective character. Furthermore, our sample size of 300 patients was rather small, in particular with regard to overall low rates of major bleedings following liver biopsy. Therefore, further validation of our data should be the subject of larger cohorts. In our institutional setting, a randomized trial of tract plugging in the presence of active bleeding from the biopsy site was not justifiable, though it would be an ideal study design to test the efficiency and safety of gelatin sponge slurry. Some post-procedural complications might not be reported to us due to follow-up treatment in another institution, especially in patients with a more distant place of residence. The amount of gelatin sponge slurry applied can only be estimated. Only the use of gelatin sponge slurry, not the amount was documented. To our knowledge, no comparative study between plugged and unplugged biopsy tract exists, therefore this study addresses an important gap of knowledge.

## Conclusion

Biopsy tract plugging with gelatin sponge slurry is a technically easy and safe procedure that can prevent major bleeding events following biopsy procedures of the liver.
